# Asymptomatic T prolymphocytic leukemia: case report and literature review

**DOI:** 10.31744/einstein_journal/2025RC1899

**Published:** 2025-10-30

**Authors:** Luiz Frederico Bezerra Honorato, Elizabeth Xisto Souto, Roberta Maria da Silva Oliveira Safranauskas, Renata Kiyomi Kishimoto, Laiz Cameirao Bento, Marilia Sandoval Passaro, Rodrigo Seiti Kojima, Nydia Strachman Bacal, Barbara Ferreira Cordeiro Galvão, Luiz Gustavo Ferreira Cortês, Elvira Deolinda Rodrigues Pereira Velloso, Nelson Hamershlak

**Affiliations:** 1 Hospital Israelita Albert Einstein São Paulo SP Brazil Hospital Israelita Albert Einstein, São Paulo, SP, Brazil.

**Keywords:** Leukemia, prolymphocytic, T-cell, T-Lymphocytes, Asymptomatic diseases

## Abstract

T-cell prolymphocytic leukemia is a rare and aggressive mature T-cell malignancy that usually presents with marked lymphocytosis, hepatosplenomegaly, lymphadenopathy, and B symptoms. However, a minority of patients present with an indolent, asymptomatic form. Case Report: A 44-year-old man was diagnosed with asymptomatic T-cell prolymphocytic leukemia after routine blood tests revealed persistent lymphocytosis. Immunophenotyping revealed a mature CD4^-^/CD8^+^ T-cell population. Cytogenetic analysis showed 14q11.2 abnormalities with TCRAD rearrangement by fluorescent in situ hybridization. A monoclonal T-cell population was confirmed by flow cytometry and polymerase chain reaction, and a *STAT5B* mutation was identified by next-generation sequencing. The patient had no cytopenia or organ involvement and a watch-and-wait strategy was adopted. The pathogenesis of T-cell prolymphocytic leukemia involves recurrent genetic alterations, including *TCL1A* rearrangements and *ATM* mutations, which promote genomic instability. Despite their aggressive nature, up to 30% of cases initially follow an indolent course, allowing for observation rather than immediate treatment. Standard therapies include alemtuzumab-based regimens and hematopoietic stem cell transplantation, although relapse rates remain high. Conclusion: This case underscores the need to recognize indolent presentations of T-cell prolymphocytic leukemia that may be managed conservatively. Further research is required to identify prognostic markers and optimize therapeutic strategies.

## INTRODUCTION

T-cell prolymphocytic leukemia (T-PLL) is a rare hematologic malignancy that accounts for approximately 2% of mature lymphoid neoplasms and was first described in 1973. It is characterized by the clonal proliferation of small- to medium-sized prolymphocytes with a mature post-thymic T-cell phenotype, typically involving the juxtaposition of *TCL1A* or *MTCP1* at the T-cell receptor locus.^(1–2)^

T-cell prolymphocytic leukemia typically presents with marked lymphocytosis (>100,000/μL), anemia, thrombocytopenia, hepatosplenomegaly, generalized, nonbulky lymphadenopathy, and B symptoms. Additional manifestations may include skin involvement, serous effusions (pleural, peritoneal), and more rarely, central nervous system involvement, with the latter occurring in only approximately 10% of patients. The skin is the most commonly affected extramedullary site and is often associated with a high tumor burden.^(2–4)^

The median interval from diagnosis to treatment initiation is typically approximately two months.^(2,5–7)^ Approximately 30% of patients initially present with an indolent and asymptomatic phase, which often progresses to active disease within 1-2 years.

With an estimated incidence of 0.6-2 cases per million annually, the disease primarily affects adults aged over 30 years, with a median age of 65 years.^(4)^ Due to its rarity, the characterization of clinical subtypes and therapeutic strategies remains limited, with most data derived from case reports or small series.

This manuscript describes a case of asymptomatic T-PLL with minimal laboratory abnormalities that did not require immediate treatment. Reporting rare presentations is essential to broaden our understanding of this rare disease and its clinical heterogeneity.

## CASE REPORT

**Figure 1:**
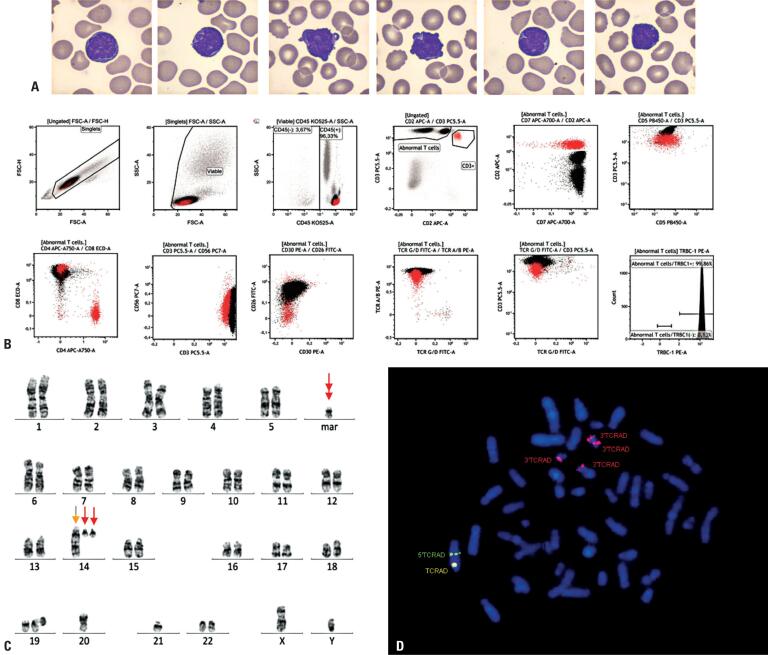
Morphologic, immunophenotypic, cytogenetic, and molecular features of indolent T-cell prolymphocytic leukemia at diagnosis

A 44-year-old man with a history of lymphocytosis that developed in June 2021 was referred for evaluation after routine blood tests revealed progressive leukocytosis. The patient remained asymptomatic with no cytopenia and a lymphocyte count of 8,000/mm^3^ (Figure 1A).

(A) Peripheral blood smear showing the presence of small to medium-sized cells with irregular nuclear contours, evident round to oval nucleoli, basophilic cytoplasm, and cytoplasmic blebs (Leishman, 630 magnification). B) Diagnostic histograms of immunophenotyping of Peripheral Blood cells by flow cytometry. 62.79% of cells were identified expressing CD2, CD3, CD5, CD7, CD8, CD26, and TCRalpha/beta. This population is negative for CD4, CD30, CD56, and TCR gamma/delta. Monoclonality was observed in this population through the analysis of TRBC1 antigen expression. The pink is the normal T lymphoid population, and the black is the clonal population. C) Image representative of karyotype of Peripheral blood cells, the composite complex clone was described as: 46~47,XY,+8,t(14;14)(q11.2;q32),+der(14)t(14;14),add(19)(p13.3),-20,add(20)(p11.2),+mar[cp17]/46,XY[3] (GTW banding). D) Metaphase fluorescence in situ hybridization (FISH) study in Peripheral blood cells using TCRA/D Break Apart probe (MetaSystems, Germany) consists of an red probe hybridizing proximal to the TCRA/D gene region at 14q11.2 and a green probe hybridizing distal to TCRA/D gene region at 14q11.2, the image shows green sign (3’TCRAD) and four red marks (5’TCRAD) suggesting gene rearrangement TCRA/D [ish(5’TCRADx5,3’TCRADx2)(5’TCRAD con 3’TCRADx1)]

Peripheral blood immunophenotyping revealed 62.7% abnormal, monoclonal T lymphoid cells, expressing CD2 (partial), CD3, CD5, CD7, CD8, CD26, and T-cell receptor (TCR) α/β, and negative for CD4, CD30, CD56, and TCR γ/δ. Monoclonality was confirmed by flow cytometry for TRBC1 antigen expression (Figure 1B), and clonal TCR rearrangement was confirmed by reverse transcription polymerase chain reaction.

Positron emission tomography-computed tomography revealed no abnormal glycolytic hypermetabolism or visceromegaly. Cytogenetic and molecular analyses were then performed. Unstimulated peripheral blood metaphase showed a complex karyotype, including a translocation between the long arms of chromosome 14 and its homolog (Figure 1C). Fluorescent in situ hybridization with a TCRAD Break Apart probe (MetaSystems^®^, Germany) confirmed *TCRAD* gene rearrangement on chromosome 14, with additional 5′ *TCRAD* copies in a marker chromosome (Figure 1D).

Molecular analysis identified a pathogenic *STAT5B* mutation (c.1924A>C, p.Asn642His) with a variant allele frequency of 13.9% along with a germline heterozygous pathogenic variant in *MLH1* (c.1731G>A, p.Ser577=), which was confirmed as a germline mutation in a fibroblast culture after a skin biopsy.

The diagnosis of T-PLL was based on the presence of clonal lymphocytosis >5×10^9^/L, T-cell clonality, *TCRAD* rearrangement, and *STAT5B* mutation. The patient remained completely asymptomatic without cytopenia or other hematologic abnormalities, and active surveillance was initiated.

This case study was approved by the Research Ethics Committee of *Hospital Israelita Albert Einstein* (CAAE: 57507322.6.0000.0071; # 5.716.523), and written informed consent was obtained from the patient.

## DISCUSSION

The pathogenesis of T-PLL involves rearrangement of the TCR, located on chromosome 14q) and juxtaposition with T-cell leukemia (TCL) genes, leading to the aberrant expression of *TCL1A, TCL1B,* or *MTCP1* oncogenes in approximately 95% of cases. *ATM* involvement and genomic instability, due to deletions or mutations that impair DNA repair mechanisms, have also been reported, albeit less frequently. Mutations in the *JAK1, JAK3,* and *STAT5B* pathways (as observed in our case) are common, resulting in constitutive activation of cellular growth signaling.^(1,3,7,13)^ Additional alterations include mutations in *EZH2, FBXW10,* and *CHEK2*. Cytogenetically, inv(14)(q11q32) or t(14;14)(q11;q32) and chromosome 8 abnormalities are frequent, with the latter leading to increased *C-MYC* expression and accelerated proliferation.^(1–5,7,8)^

Diagnosis is typically established through analysis of the peripheral blood or bone marrow. However, a bone marrow biopsy is often unnecessary as the diagnosis can generally be confirmed through peripheral blood examination.^(2,5,8,9)^ Although most patients present with aggressive features, approximately 30% show an indolent, asymptomatic course, with a median time to progression of 1-2 years, and occasional reports of progression of up to 4 years.^(10)^

Diagnostic criteria include T-cell lymphocytosis >5×10^9^/L, a T-PLL immunophenotype in peripheral blood or bone marrow, and clonal T-cell population confirmed by polymerase chain reaction or flow cytometry, in association with chromosome 14 abnormalities or overexpression of *TCL1* or *MTCP1*. Alternatively, if the first two major criteria are satisfied, a diagnosis can be established using at least one minor criterion.^(4)^

T-cell prolymphocytic leukemia presents with three morphological variants. The most common type (50-75% of cases) consists of small- to medium-sized lymphocytes with a high nuclear-to-cytoplasmic ratio, condensed chromatin, and a single prominent nucleolus. The second group (20-25% of cases) has atypical lymphocytes with convoluted nuclei, resembling Sézary cells. The third variant is characterized by small atypical lymphocytes that mimic chronic lymphocytic leukemia.^(2)^ The immunophenotype corresponds to post-thymic mature T-cells, typically TdT and CD1a negative, and CD3 positive. The most frequent phenotype is CD4^+^/CD8^–^ (40-60%), followed by CD4^+^/CD8^+^ (25-41%) and CD4^–^/CD8^+^ (15%). CD52 is commonly expressed, making it therapeutically relevant. The most common TCR is αβ.^(11,12)^ Our patient had the rare CD4^–^/CD8^+^ phenotype with gamma-delta TCR expression.

One of the largest T-PLL studies included 119 patients (75 untreated and 43 previously treated) between 1990 and 2016, with a mortality rate of 80%. Poor prognostic indicators included pleural effusion, *TCL1* positivity, leukocytosis >200,000/μL, anemia, elevated LDH (>1669IU/L), and non-Caucasian ethnicity. A complex karyotype is observed in up to 80% of the cases and is considered a negative prognostic factor. Other poor prognostic factors include an age older than 65 years, serous effusion, central nervous system or hepatic involvement, bulky lymphadenopathy, high *TCL1* expression, bone marrow failure, and organ dysfunction.^(1,7,13–15)^

The main differential diagnoses were adult T-cell leukemia/lymphoma, Sézary syndrome, large granular lymphocytic leukemia, T/B-cell acute lymphoblastic leukemia, hepatosplenic lymphoma, chronic lymphocytic leukemia, and mantle cell lymphoma. Diagnosis requires integration of clinical presentation, immunophenotyping, and cytogenetics.

There is no solid evidence to support the early treatment of asymptomatic patients. Patients who respond poorly to chemotherapy should be managed based on clinical observations. The T-PLL International Study Group (TPLL-ISG) recommends initiating therapy in patients with fatigue, B symptoms, bone marrow failure, symptomatic adenopathy, hepatosplenomegaly, rapidly increasing lymphocytosis, or significant extranodal involvement.^(4,12)^

Most chemotherapy regimens are based on CHOP or FMC, yielding complete responses lasting only 3-6 months, and a median overall survival of 8-9 months.^(2,6,10,12)^ Better outcomes were associated with the use of nonchemotherapeutic agents. Pentostatin, an ADA-inhibiting antimetabolite, has shown modest efficacy, either as a monotherapy or in combination. In responders, the overall survival can reach 16-18 months, although the responses are typically short, with a median duration of six months.^(6,10,12)^

Alemtuzumab, a monoclonal antibody against CD52, has shown the most promising clinical response in T-PLL, whether administered as induction, monotherapy, combination, salvage, or maintenance therapy. It is generally well tolerated with manageable side effects, including infusion reactions, lymphopenia, and infections.^(15–19)^ The intravenous formulation resulted in higher response and survival rates than the subcutaneous version.^(17,18, 20)^

A retrospective study of 119 patients showed that 55 previously untreated patients received alemtuzumab-based regimens with 42 receiving monotherapy and 13 receiving alemtuzumab in combination with pentostatin. The overall response and complete response rates for alemtuzumab alone were 83% and 66%, respectively, and those for combination therapy were 82% and 73%, respectively. In a phase II study involving 13 patients, the overall response rate, progression-free survival, and overall survival rates were 69%, 8 months, and 10 months, respectively, with alemtuzumab plus pentostatin.^(7,15)^

Alemtuzumab also demonstrated utility as a salvage therapy, with a study of 37 previously treated patients reporting an overall response rate of 76% and a median overall survival of 10 months.^(16,21)^ It can be used for maintenance therapy after chemotherapy to improve responses in patients who are not initially eligible.^(18,19)^

Given the aggressiveness of T-PLL, remission is often short and relapse is common. Allogeneic stem cell transplantation should be considered for patients who achieve a complete response, particularly as first-line therapies. Autologous transplantation may be an option in cases without a suitable donor or in those who are ineligible for allogeneic transplantation. One-to-four-year survival rates range from 20-40%, with transplant-related mortality and relapse rates of approximately 30-40%.^(21–27)^

## CONCLUSION

T-cell prolymphocytic leukemia is a rare disease that typically presents aggressively and is associated with a high risk of relapse. In symptomatic cases, treatment is based on immunotherapy, either alone or in combination, followed by allogeneic hematopoietic stem cell transplantation during the first remission. However, this strategy is associated with high morbidity and frequent relapse, emphasizing the limitations of the current treatments.

Our patient belonged to the indolent subgroup, remained asymptomatic, and did not require immediate treatment. The selected strategy was based on clinical observations. Due to its rarity, increasing awareness through case reports and discussions is essential to stimulate debate and enhance our understanding of this challenging malignancy.
